# In Search of an Accurate Evaluation of Intrahepatic Cholestasis of Pregnancy

**DOI:** 10.6064/2012/496489

**Published:** 2012-08-01

**Authors:** Manuela Martinefski, Mario Contin, Silvia Lucangioli, Maria Beatriz Di Carlo, Valeria Tripodi

**Affiliations:** ^1^Department of Analytical Chemistry and Physicochemistry, Faculty of Pharmacy and Biochemistry, University of Buenos Aires, Junin 956, 1113 Buenos Aires, Argentina; ^2^Consejo Nacional de Investigaciones Científicas y Tecnológicas, CONICET, Argentina; ^3^Department of Pharmaceutical Technology, Faculty of Pharmacy and Biochemistry, University of Buenos Aires, Junin 956, 1113 Buenos Aires, Argentina; ^4^Department of Clinical Biochemistry, Faculty of Pharmacy and Biochemistry, University of Buenos Aires, Junin 956, 1113 Buenos Aires, Argentina

## Abstract

Until now, biochemical parameter for diagnosis of intrahepatic cholestasis of pregnancy (ICP) mostly used is the rise of total serum bile acids (TSBA) above the upper normal limit of 11 *μ*M. However, differential diagnosis is very difficult since overlapped values calculated on bile acids determinations, are observed in different conditions of pregnancy including the benign condition of pruritus gravidarum. The aim of this work was to determine the better markers in ICP for a precise diagnosis together with parameters associated with severity of symptoms and treatment evaluation. Serum bile acid profiles were evaluated using capillary electrophoresis in 38 healthy pregnant women and 32 ICP patients and it was calculated the sensitivity, specificity, accuracy, predictive values and the relationships of certain individual bile acids in pregnant women in order to replace TSBA determinations. The evaluation of the results shows that LCA and UDCA/LCA ratio provided information for a more complete and accurate diagnosis and evaluation of ICP than calculation of solely TSBA levels in pregnant women.

## 1. Introduction

Intrahepatic cholestasis of pregnancy (ICP) is a pregnancy-specific liver disease and it takes place in the second or third trimester of gestation and it spontaneously disappears after delivery [1, 2].

ICP is characterized by generalized skin pruritus and abnormal liver function, and it is associated with increased fetal distress, premature deliveries, and perinatal mortality and morbidity. Therefore, an early and accurate diagnosis of a risky pregnancy produced by ICP is essential [3–5].

Usually, diagnosis of ICP is based on pruritus with mild or moderate elevated levels of amino transferases and/or raised total serum bile acids (TSBAs) [6]. However, it is often difficult to accomplish an accurate diagnosis by performing solely routine laboratory tests because they are also altered in some other conditions of pregnant women. In fact, the existence of subclinical cholestasis during pregnancy may also impair the identification of the disease. Moreover, pruritus in pregnancy is a common symptom but it could be the only evidence in ICP, so it is necessary to discriminate women with ICP from those with benign condition of pruritus gravidarum [5, 7]. Furthermore, a subgroup of asymptomatic pregnant women with high levels of TSBA, and normal liver function tests but not showing pruritus, has recently been classified as asymptomatic hypercholanemia of pregnancy (AHP) [7–10].

In agreement with some authors [4, 5, 7, 11], we have reported that the individual serum bile acid determinations provide more information than TSBA determination, though the interpretation of a complete serum bile acid profile could be difficult to establish because clinicians are accustomed to evaluate ICP using one single value (TSBA) as determinant instead of interpreting many values derived from the analysis of individual bile acids.

Several reports have demonstrated that different ratios calculated from individual bile acid determinations are useful in the comprehension of different types of liver diseases [2, 12–14]. However, a latter report has recently determined that calculation of cholic acid/chenodeoxycholic acid (CA/CDCA) ratio contributes little to ICP diagnosis [15].

Otherwise, several authors have demonstrated that lithocholic acid (LCA) is toxic [16–19] and ursodeoxycholic acid (UDCA) posseses protective effects [18, 20, 21]. Thus, a convenient proportion between both bile acids could be an interesting indicator to precisely diagnose ICP and/or to conveniently manage a medical treatment of the disease.

Taking into account the need for better markers for the precise diagnosis of ICP and the search of parameters associated with severity of symptoms and treatment efficiency, we determined the sensitivity, specificity, accuracy, predictive values, and the relationships of certain individual bile acids in pregnant women to replace TSBA determinations.

## 2. Patients and Methods

### 2.1. Settings and Study Design

The study was conducted in normal pregnant women and patients with ICP at the Hospital de Clinicas “Jose de San Martin” of the city of Buenos Aires, associated to the University of Buenos Aires. This study was performed according to the principles of the Declaration of Helsinki and it was approved by the Institutional Review Board and Bioethical Committee of our Institution. Written consent was obtained in every case.

### 2.2. Patients

During the study period, 38 healthy pregnant women and 32 ICP patients in the third trimester of pregnancy were studied. Diagnosis of ICP was based on the presence of pruritus observed in patients and/or at least one of the two aminotransferases like alanine-aminotransferase (ALT) or aspartate-aminotransferase (AST), levels above the upper normal limits (40 and 31 UI/L, resp.), during the second half of an otherwise uneventful pregnancy, and their control with normalization of cholestasis after delivery were considered. In addition, we took in account the absence of infections by hepatitis viruses like HAV, HBV or HCV, autoimmune diseases, moderate (≤2 drinks/day) to severe alcohol intake, HIV infection, skin diseases, or biliary obstruction evaluated by liver ultrasound at the moment of the recruitment. Twin-birth cases were excluded.

Pruritus was arbitrarily measured in an ordinary scale: grade 1 intermittent, nocturnal, and slight; grade 2, continuous diurnal, and nocturnal, from slight to moderate; grade 3 severe and grade 4 severe but also accompanied with insomnia or itching lesions.

Severity of symptoms was determined by clinicians according to pruritus score and levels of aminotransferases. The lowest pruritus score with low levels of aminotransferases in serum was the least severe grade of ICP disease.

From 32 ICP patients, 23 had high pruritus score (grade 3/4) and were only treated with UDCA (900 mg/day). Two serum samples were taken within a 15-day period: the first was obtained before treatment and the second was obtained 15 days after the beginning of UDCA treatment.

Serum samples from 9 ICP patients with low pruritus score and without treatment were also taken for comparison during the period of study.

Additional clinical data of the patients studied are shown in Table 1.

## 3. Methods

### 3.1. Liver Function Tests

Serum samples were obtained after a fasting period of eight hours, and aliquots were frozen at –20°C before bile acid determinations. Alanine-aminotransferase (ALT), aspartate-aminotransferase (AST), alkaline phosphatase (ALP), gamma-glutamyltranspeptidase (*γ*-GT), 5′nucleotidase (5′NT) activities, and total bilirubin concentrations were carried out by routine automated techniques.

### 3.2. Total Serum Bile Acids and Serum Bile Acid Profile

Total serum bile acid (TSBA), cholic acid (CA), chenodeoxycholic acid (CDCA), deoxycholic acid (DCA), lithocholic acid (LCA), and ursodeoxycholic acid (UDCA) in their free, glycine, and taurine derivative forms were assessed by capillary electrophoresis. A detailed description of the analytical method performed in this study has been described by Tripodi et al. [10]. Briefly, simultaneous determinations of 15 serum bile acids were performed using an off-line C18 solid phase extraction procedure for sample cleanup and concentration. This step was followed by the complete separation of the bile acids using cyclodextrin-modified micellar electrokinetic chromatography with UV detection, and quantitation was accomplish in less than 12 min. The same determinations were taken in sera from patients and controls for comparison.

### 3.3. Sensibility, Specificity, Accuracy, and Predictive Values

In order to evaluate the better marker for the complete diagnosis and followup of the disease, sensitivity, specificity, accuracy, positive predictive values (PPV), and negative predictive values (NPV) were calculated xfor each marker.

### 3.4. Statistical Analysis

Shapiro-Wilksś *W* test of normality was performed. Kruskal-Wallis nonparametric analysis followed by the Mann-Whitney *U*-test was also used. Differences between groups were analyzed by Student's *t*-test or nonparametrical tests, according to distribution. Spearman *r* coefficient was calculated for correlations. Levels of significance were established at *P* < 0.05 and ROC curves were used to determine cut-off levels.

## 4. Results and Discussion

### 4.1. Liver Function Tests

No difference in routine liver function tests of ICP women with low pruritus and normal pregnancies was observed. However, traditional biochemical parameters such as determinations of the levels of total bilirubin, AST, ALT, and ALP were increased in ICP women with high pruritus score (Table 1). Taking into account that pruritus in pregnancy is a common symptom, it was not possible to distinguish patients with low grade of ICP from those with the benign condition of pruritus gravidarum based only on pruritus and traditional liver function tests.

### 4.2. TSBA and Serum Bile Acid Profiles

On the basis of the serum bile acid analysis, we could differentiate a healthy subgroup of patients with normal clinical and biochemical features showing TSBA levels above the cut-off value of 11 *μ*M (TSBA = 19.4 ± 2.0 *μ*M, mean ± SEM, *n* = 15) and they were classified as AHP.

**Figure 1:**
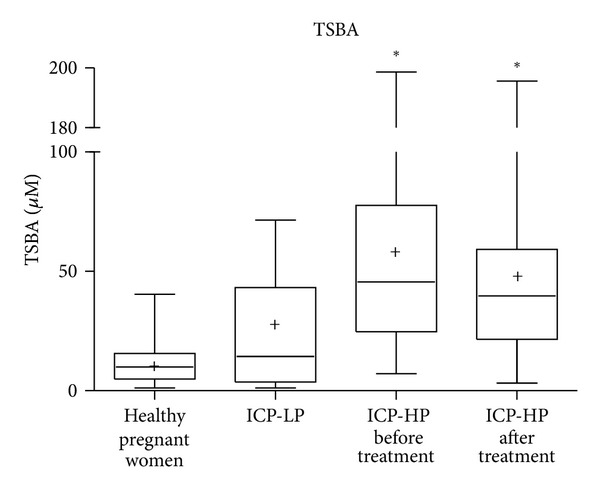
TSBA levels in the groups studied: healthy pregnant women, ICP patients with low pruritus score (ICP-LP) and ICP patients with high pruritus score (ICP-HP) before and after treatment. *P < 0.0001 respect to healthy pregnant women.

Women with ICP had significantly higher levels of TSBA than clinically healthy pregnant women (Table 2). Nonetheless, in previous reports [7, 11] we have just observed that, in some cases, TSBA values were overlapped in both groups. On the basis of TSBA levels, for comparison, it was not possible to distinguish the results of ICP patients with low pruritus from those calculated in normal pregnant women. No significant difference between mean values of solely TSBA determination in ICP patients with high pruritus score before and after treatment was found (Figure 1).

UDCA levels were high in AHP patients with respect to normocholanaemic pregnant women and to all ICP patients before treatment. Moreover, UDCA levels were found to overlap in ICP patients showing different pruritus scores before treatment and patients with normocholanemia (Table 2). Taking solely UDCA as the parameter of diagnosis, it was not possible to recognize different grades of ICP severity or to differentiate between nontreated ICP patients and normocholanaemic patients.

Table 2 also shows increased LCA levels in women with ICP with respect to those in the AHP and normocolanaenic groups in agreement with the severity of the pruritus symptom (*r* = 0.448, *P* < 0.02). After UDCA therapy, LCA levels decreased in all patients showing a lower mean value than in ICP patients with low pruritus score, although never reaching normal levels.

It was also observed that CDCA and CA levels were increased in ICP patients with high pruritus score despite UDCA treatment. DCA levels were high in patients with high pruritus score, but the mean value was not statistically different with respect to the mean value of AHP patients. A comparison DCA mean value in healthy pregnant women against ICP patients showed no significant difference between both groups (3.0  ±  0.8 *μ*M versus 7.7  ±  2.5 *μ*M, mean ± SEM). From these calculations, DCA did not prove to be a useful value to diagnose ICP. In the same sense, the results of CA/CDCA ratio presented a high degree of dispersion making difficult to separate the groups of normal subjects and ICP patients.

An inspection of UDCA/LCA ratios showed that the high values observed in AHP patients could be attributed to the increment of UDCA levels in this class of patients. It could be possible to hypothesize that this high ratio comes from a protective effect of UDCA levels in AHP patients not showing ICP symptoms even with elevated TSBA in serum. These evidences will be demonstrated in further studies. Moreover, UDCA/LCA ratios in patients with low and high pruritus scores before treatment were lower than AHP (*P* < 0.001) and normal pregnancies (*P* < 0.0001). Likewise, after treatment, all ICP patients showed a dramatic increase in UDCA/LCA ratio (*P* < 0.0001).

## 5. Diagnosis and Prediction of ICP through Calculation of Sensitivity, Specificity, Accuracy, and Predictive Values

### 5.1. Determination of Bile Acids Cut-Off Levels

The cut-off levels for normality, determined by ROC curves, were LCA: <1.1 *μ*M, UDCA/LCA ratio: >1.0, UDCA: >2.3 *μ*M, CDCA: <3.6 *μ*M, CA: <4.2 *μ*M, and DCA <1.3 *μ*M. The cut-off level of TSBA widely used is <11 *μ*M. As CA/CDCA ratio did not show significative difference between controls and ICP patients, cut-off levels were not calculated in this work.

### 5.2. Differential Diagnosis

**Figure 2:**
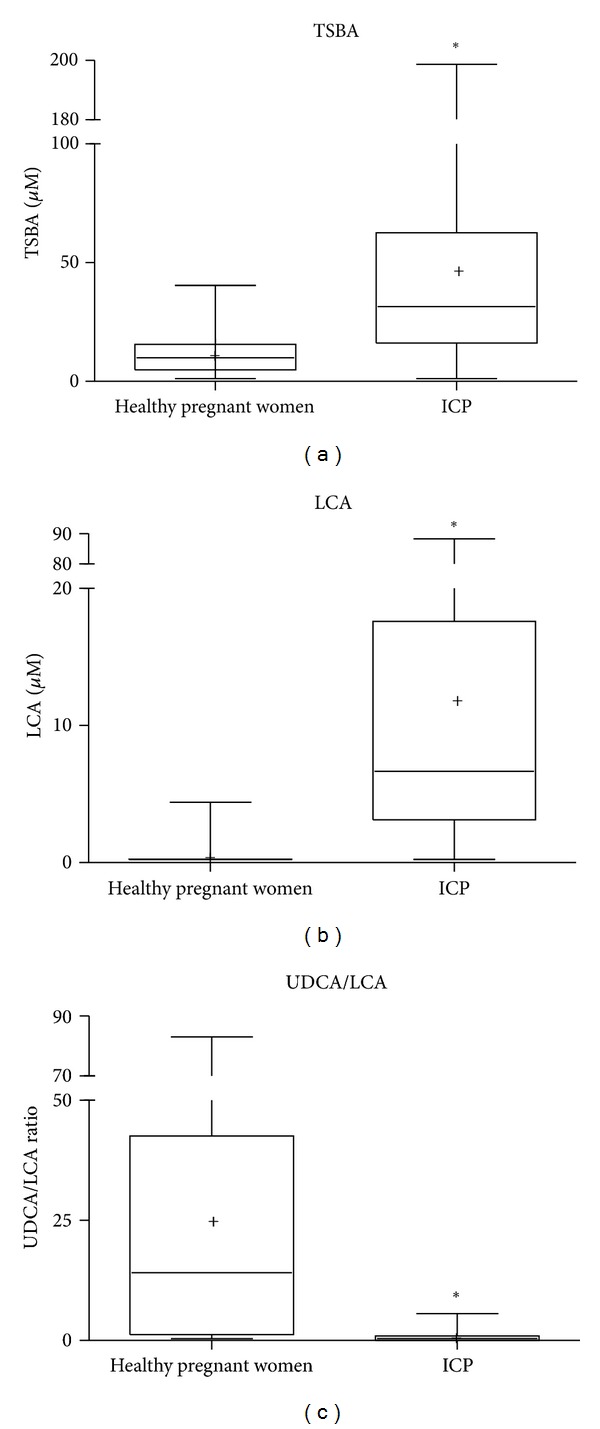
Differential diagnosis comparing TSBA levels (a), LCA levels (b), and UDCA/LCA ratio (c) in ICP and healthy pregnant women. *P < 0.0001 with respect to healthy pregnant women.

In order to differentiate patients suffering the disease, independently of the grade of severity of the symptoms, the following parameters were calculated: sensitivity, specificity, accuracy, PPV, and NPV from determination of total bile acids and display of their profiles in sera taken from all pregnants under study. It was observed that LCA value and UDCA/LCA ratio provided the best evaluation parameters. In this sense, the highest sensitivity (84%) and NPV (88%) was achieved by determination of LCA level, the highest specificity (100%) and PPV (100%) from calculation of UDCA/LCA ratio, and the highest accuracy (91.5%) if both LCA and UDCA/LCA ratio were taken into account. In the same cases, values of TSBA only showed 81% sensitivity, 61% specificity, 63% PPV, 79% PPN, and 70% accuracy.  Figure 2 illustrates a comparison of TSBA levels (Figure 2(a)), LCA levels (Figure 2(b)), and UDCA/LCA ratio (Figure 2(c)) between healthy pregnants and ICP groups.

In agreement with Huang et al. [15], we could not find that calculation of CA/CDCA ratio can provide additional information for better diagnosis of ICP. Mean values of DCA determinations showed no significant difference between healthy pregnants and ICP patients and they could not be taken as a useful indicator.

### 5.3. Severity of the Symptoms

Although pruritus is a subjective symptom, the physicians are accustomed to associate greater severity and impaired outcome of the disease with a higher degree of pruritus score. In order to determine a more accurate predictor of ICP severity, a correlation between severity of symptoms and individual bile acid determinations in sera was found. In this study, LCA values showed the best correlation (*r* = 0.448, *P* < 0.02).

In ICP patients, increased LCA levels were related to the severity of the symptoms and they decreased after UDCA therapy, although never reaching values of healthy pregnant women. Therefore, LCA is taken as a limited value in clinical studies unless its levels can be compared before and after treatment and the decrease of the values hs been confirmed after therapy.

### 5.4. Effectiveness of Treatment

Assuming that the effectiveness of treatment is reflected in the correction of the biochemical parameters and pruritus improvement, we examined whether there is a significant difference in the mean values of LCA, DCA, UDCA/LCA ratio, and TSBA levels before and after therapy and correlations with symptoms. In Table 2, it was observed that TSBA levels remained elevated in all ICP-treated patients (51.6 ± 13.3 *μ*M versus 50.9 ± 12.3 *μ*M) probably due to the treatment that produces an increment UDCA concentrations.

LCA is dramatically reduced after therapy in all patients (20.3 ± 5.6 *μ*M versus 2.2 ± 1.0 *μ*M, *P* < 0.0001), although not reaching levels below the cut-off value. However, UDCA/LCA ratio is highly increased above the cut-off level (0.59 ± 0.37 *μ*M versus 79.9 ± 28.8 *μ*M, *P* < 0.0001). It was also observed a significant decrease in DCA values in 74.1% of all treated patients.

The symptomatic relief in pruritus in ICP treated patients highly correlated with the fall in LCA levels (*r* = 0.996) and also with the increase of UDCA/LCA ratio (*r* = 0.822). However, there was no correlation between pruritus score and DCA or TSBA after treatment.

If the purpose is to evaluate the effectiveness of treatment, LCA levels and UDCA/LCA ratio proved to be the best corrected parameters.

## 6. Conclusions

Values of LCA together with UDCA/LCA ratio are advisable to perform a complete diagnostic evaluation and evolution after treatment of ICP. As markers of ICP, they provide more accurate values than TSBA or any other individual bile acid determination.

## Figures and Tables

**Table 1:** Clinical and biochemical characteristics of women with ICP and women with normal pregnancies.

	Groups studied		
	Normal pregnancies	ICP (low pruritus score)	ICP (high pruritus score)
Number of subjects	38	9	23
Age in years (range)	27 (23–39)	31 (24–39)	27 (21–33)
Number of pregnancies (*n*)	1.8	1.9	2.3
History of ICP (*n*)	0	5	5
Familiar history of ICP (*n*)	0	5	5
Total bilirubin (mg/mL)	0.40 ± 0.02	0.40 ± 0.03	0.83 ± 0.08*
AST (IU/L)	21.0 ± 1.5	21.0 ± 3.0	92.0 ± 13.2**
ALT (IU/L)	14.4 ± 0.8	17.0 ± 2.7	128 ± 25**
ALP (IU/L)	313 ± 20	282 ± 56	674 ± 50**
*γ*-GT (IU/L)	38 ± 9	36 ± 11	33 ± 3
5′NT (IU/L)	7.8 ± 1.9	7.2 ± 2.4	7.9 ± 2.6

Results are expressed as means ± SEM. For total bilirubin, the normal range is up to 1.1 mg/dL. The normal ranges for AST, ALT, and ALP are 12–31, 12–40, and 90–240 IU/L, respectively. For *γ*-GT and 5′NT, the normal ranges are up to 40 and 9.7 IU/L, respectively.

^∗^ *P* < 0.05 and ^∗∗^ *P* < 0.01 compared with normal pregnancies.

**Table 2:** Comparison of TSBA levels and serum bile acid profiles in all of the groups studied.

	Normocholanaemic pregnant women (*n* = 23)	AHP (*n* = 15)	ICP low pruritus score (*n* = 9)	ICP high pruritus score (*n* = 23)	ICP high pruritus score treated with UDCA (*n* = 23)
TSBA (*μ*M)	5.8 ± 0.7	19.4 ± 2.0^§§^	24.2 ± 8.3	54.9 ± 12.2^§§^	47.6 ± 11.9^§§^
UDCA (*μ*M)	3.1 ± 0.7	10.8 ± 1.4^§§^	1.1 ± 0.5	5.2 ± 2.8	28.3 ± 6.3^§§,a^
LCA (*μ*M)	0.34 ± 0.06	0.60 ± 0.28	6.5 ± 3.6^§^	20.3 ± 5.6^§§^	2.2 ± 1.0^∗,a^
CDCA (*μ*M)	0.75 ± 0.24	1.3 ± 0.7	1.8 ± 1.0	10.4 ± 4.0^∗∗^	8.8 ± 5.6
CA (*μ*M)	0.40 ± 0.17	1.1 ± 0.5	8.8 ± 5.2^∗∗^	7.1 ± 2.3^§^	5.7 ± 1.9^§^
DCA (*μ*M)	1.9 ± 0.6	5.6 ± 1.6^§^	6.0 ± 4.3^∗^	12.0 ± 4.6^§^	2.7 ± 1.3^§,b^
CA/CDCA	1.8 ± 0.9	5.5 ± 2.7	7.7 ± 3.9	18.5 ± 10.9	13.7 ± 7.5
UDCA/LCA ratio	11.1 ± 2.9	41.5 ± 7.5^§^	0.65 ± 0.23^§^	0.59 ± 1.4^§§^	79.9 ± 28.8^∗∗,a^

Results are expressed as means ± SEM. Bile acids are expressed in their free, glycine, and taurine forms. ^∗^ *P* < 0.05^∗∗^ *P* < 0.01, ^§^ *P* < 0.001 and ^§§^ *P* < 0.0001, with respect to normocholanaemic pregnant women. ^a^ *P* < 0.0001 and ^b^ *P* < 0.05 with respect to ICP high pruritus score.

## References

[1] Shaw D, Frohlich J, Wittmann BAK, Willms M (1982). A prospective study of 18 patients with cholestasis of pregnancy. *American Journal of Obstetrics and Gynecology*.

[2] Brites D, Rodrigues CMP, Oliveira N, Cardoso MDC, Graça LM (1998). Correction of maternal serum bile acid profile during ursodeoxycholic acid therapy in cholestasis of pregnancy. *Journal of Hepatology*.

[3] Diaferia A, Nicastri PL, Tartagni M, Loizzi P, Iacovizzi C, Di Leo A (1996). Ursodeoxycholic acid therapy in pregnant women with cholestasis. *International Journal of Gynecology and Obstetrics*.

[4] Bacq Y, Myara A, Brechot MC (1995). Serum conjugated bile acid profile during intrahepatic cholestasis of pregnancy. *Journal of Hepatology*.

[5] Meng LJ, Reyes H, Palma J, Hernandez I, Ribalta J, Sjövall J (1997). Effects of ursodeoxycholic acid on conjugated bile acids and progesterone metabolites in serum and urine of patients with intrahepatic cholestasis of pregnancy. *Journal of Hepatology*.

[6] Reyes H, Simon FR (1993). Intrahepatic cholestasis of pregnancy: an estrogen-related disease. *Seminars in Liver Disease*.

[7] Castaño G, Lucangioli S, Sookoian S (2006). Bile acid profiles by capillary electrophoresis in intrahepatic cholestasis of pregnancy. *Clinical Science*.

[8] Lunzer M, Barnes P, Byth K, O’Halloran M (1986). Serum bile acid concentrations during pregnancy and their relationship to obstetric cholestasis. *Gastroenterology*.

[9] Pascual MJ, Serrano MA, El-Mir MY, Macias RIR, Jiménez F, Marin JJG (2002). Relationship between asymptomatic hypercholanaemia of pregnancy and progesterone metabolism. *Clinical Science*.

[10] Tripodi VP, Lucangioli SE, Scioscia SL, Carducci CN (2003). Simultaneous determination of free and conjugated bile acids in serum by cyclodextrin-modified micellar electrokinetic chromatography. *Journal of Chromatography B*.

[11] Lucangioli SE, Castaño G, Contin MD, Tripodi VP (2009). Lithocholic acid as a biomarker of intrahepatic cholestasis of pregnancy during ursodeoxycholic acid treatment. *Annals of Clinical Biochemistry*.

[12] Azer SA, Klaassen CD, Stacey NH (1997). Biochemical assay of serum bile acids: methods and applications. *British Journal of Biomedical Science*.

[13] Puls T, Beuers U (2007). Intrahepatic cholestasis of pregnancy. *Orphanet Journal of Rare Diseases*.

[14] Nakamura K, Ichimiya H, Nakayama F (1992). Alteration of bile acid metabolism in two-thirds hepatectomized rat. *Journal of Gastroenterology and Hepatology*.

[15] Huang WM, Gowda M, Donnelly JG (2009). Bile acid ratio in diagnosis of intrahepatic cholestasis of pregnancy. *American Journal of Perinatology*.

[16] Pascual MJ, Serrano MA, El-Mir MY, Macias RIR, Jiménez F, Marin JJG (2002). Relationship between asymptomatic hypercholanaemia of pregnancy and progesterone metabolism. *Clinical Science*.

[17] Roda A, Cerre C, Simoni P, Polimeni C, Vaccari C, Pistillo A (1992). Determination of free and amidated bile acids by high-performance liquid chromatography with evaporative light-scattering mass detection. *Journal of Lipid Research*.

[18] Lee BL, New AL, Ong CN (1997). Comparative analysis of conjugated bile acids in human serum using high-performance liquid chromatography and capillary electrophoresis. *Journal of Chromatography B*.

[19] Ceryak S, Bouscarel B, Malavolti M, Fromm H (1998). Extrahepatic deposition and cytotoxicity of lithocholic acid: studies in two hamster models of hepatic failure and in cultured human fibroblasts. *Hepatology*.

[20] Brites D (2002). Intrahepatic cholestasis of pregnancy: changes in maternal-fetal bile acid balance and improvement by ursodeoxycholic acid.. *Annals of Hepatology*.

[21] Copaci I, Micu L, Iliescu L, Voiculescu M (2005). New therapeutical indications of ursodeoxycholic acid. *Romanian Journal of Gastroenterology*.

